# Supplementary material for: Bayesian refinement of protein structures and ensembles against SAXS data using molecular dynamics
Source: PLoS Comput Biol. 2017 Oct 18;13(10):e1005800. doi: 10.1371/journal.pcbi.1005800 (PMC5662244; doi:10.1371/journal.pcbi.1005800)
Supplement: S1 Archive — (BZ2) [file pcbi.1005800.s011.tar.bz2 › gromacs_4.62_bayesian_saxs_refi/share/html/online/g_traj.html]

g\_traj


| g\_traj Main Table of Contents |

**VERSION 4.6  
Sat 19 Jan 2013**

---

### Description

g\_traj plots coordinates, velocities, forces and/or the box.
With -com the coordinates, velocities and forces are
calculated for the center of mass of each group.
When -mol is set, the numbers in the index file are
interpreted as molecule numbers and the same procedure as with
-com is used for each molecule.

Option -ot plots the temperature of each group,
provided velocities are present in the trajectory file.
No corrections are made for constrained degrees of freedom!
This implies -com.

Options -ekt and -ekr plot the translational and
rotational kinetic energy of each group,
provided velocities are present in the trajectory file.
This implies -com.

Options -cv and -cf write the average velocities
and average forces as temperature factors to a .pdb file with
the average coordinates or the coordinates at -ctime.
The temperature factors are scaled such that the maximum is 10.
The scaling can be changed with the option -scale.
To get the velocities or forces of one
frame set both -b and -e to the time of
desired frame. When averaging over frames you might need to use
the -nojump option to obtain the correct average coordinates.
If you select either of these option the average force and velocity
for each atom are written to an .xvg file as well
(specified with -av or -af).

Option -vd computes a velocity distribution, i.e. the
norm of the vector is plotted. In addition in the same graph
the kinetic energy distribution is given.

### Files

| option | filename | type | description |
| --- | --- | --- | --- |
| **-f** | traj.xtc | Input | Trajectory: xtc trr trj gro g96 pdb cpt |
| **-s** | topol.tpr | Input | Structure+mass(db): tpr tpb tpa gro g96 pdb |
| **-n** | index.ndx | Input, Opt. | Index file |
| **-ox** | coord.xvg | Output, Opt. | xvgr/xmgr file |
| **-oxt** | coord.xtc | Output, Opt. | Trajectory: xtc trr trj gro g96 pdb cpt |
| **-ov** | veloc.xvg | Output, Opt. | xvgr/xmgr file |
| **-of** | force.xvg | Output, Opt. | xvgr/xmgr file |
| **-ob** | box.xvg | Output, Opt. | xvgr/xmgr file |
| **-ot** | temp.xvg | Output, Opt. | xvgr/xmgr file |
| **-ekt** | ektrans.xvg | Output, Opt. | xvgr/xmgr file |
| **-ekr** | ekrot.xvg | Output, Opt. | xvgr/xmgr file |
| **-vd** | veldist.xvg | Output, Opt. | xvgr/xmgr file |
| **-cv** | veloc.pdb | Output, Opt. | Protein data bank file |
| **-cf** | force.pdb | Output, Opt. | Protein data bank file |
| **-av** | all\_veloc.xvg | Output, Opt. | xvgr/xmgr file |
| **-af** | all\_force.xvg | Output, Opt. | xvgr/xmgr file |

### Other options

| option | type | default | description |
| --- | --- | --- | --- |
| **-[no]h** | bool | no | Print help info and quit || **-[no]version** | bool | no | Print version info and quit || **-nice** | int | 19 | Set the nicelevel || **-b** | time | 0 | First frame (ps) to read from trajectory || **-e** | time | 0 | Last frame (ps) to read from trajectory || **-dt** | time | 0 | Only use frame when t MOD dt = first time (ps) || **-tu** | enum | ps | Time unit: fs, ps, ns, us, ms or s || **-[no]w** | bool | no | View output .xvg, .xpm, .eps and .pdb files || **-xvg** | enum | xmgrace | xvg plot formatting: xmgrace, xmgr or none || **-[no]com** | bool | no | Plot data for the com of each group || **-[no]pbc** | bool | yes | Make molecules whole for COM || **-[no]mol** | bool | no | Index contains molecule numbers iso atom numbers || **-[no]nojump** | bool | no | Remove jumps of atoms across the box || **-[no]x** | bool | yes | Plot X-component || **-[no]y** | bool | yes | Plot Y-component || **-[no]z** | bool | yes | Plot Z-component || **-ng** | int | 1 | Number of groups to consider || **-[no]len** | bool | no | Plot vector length || **-[no]fp** | bool | no | Full precision output || **-bin** | real | 1 | Binwidth for velocity histogram (nm/ps) || **-ctime** | real | -1 | Use frame at this time for x in -cv and -cf instead of the average x || **-scale** | real | 0 | Scale factor for .pdb output, 0 is autoscale |

---

http://www.gromacs.org  
gromacs@gromacs.org
